# Mapping of pigmentation QTL on an anchored genome assembly of the cichlid fish, *Metriaclima zebra*

**DOI:** 10.1186/1471-2164-14-287

**Published:** 2013-04-27

**Authors:** Claire T O’Quin, Alexi C Drilea, Matthew A Conte, Thomas D Kocher

**Affiliations:** 1 Department of Biology, University of Maryland, College Park, MD 20742, USA

## Abstract

**Background:**

Pigmentation patterns are one of the most recognizable phenotypes across the animal kingdom. They play an important role in camouflage, communication, mate recognition and mate choice. Most progress on understanding the genetics of pigmentation has been achieved via mutational analysis, with relatively little work done to understand variation in natural populations. Pigment patterns vary dramatically among species of cichlid fish from Lake Malawi, and are thought to be important in speciation. In this study, we crossed two species, *Metriaclima zebra* and *M*. *mbenjii*, that differ in several aspects of their body and fin color. We genotyped 798 SNPs in 160 F_2_ male individuals to construct a linkage map that was used to identify quantitative trait loci (QTL) associated with the pigmentation traits of interest. We also used the linkage map to anchor portions of the *M*. *zebra* genome assembly.

**Results:**

We constructed a linkage map consisting of 834 markers in 22 linkage groups that spanned over 1,933 cM. QTL analysis detected one QTL each for dorsal fin xanthophores, caudal fin xanthophores, and pelvic fin melanophores. Dorsal fin and caudal fin xanthophores share a QTL on LG12, while pelvic fin melanophores have a QTL on LG11. We used the mapped markers to anchor 66.5% of the *M*. *zebra* genome assembly. Within each QTL interval we identified several candidate genes that might play a role in pigment cell development.

**Conclusion:**

This is one of a few studies to identify QTL for natural variation in fish pigmentation. The QTL intervals we identified did not contain any pigmentation genes previously identified by mutagenesis studies in other species. We expect that further work on these intervals will identify new genes involved in pigment cell development in natural populations.

## Background

Most vertebrate species display a complex and species-specific pigment pattern that enhances organismal fitness by contributing to crypsis, signaling, or mate recognition. Variation in pigmentation arises through differences in development [1,2], nutrition [3], and physiology [4]. Many species use neural mechanisms to rapidly alter their pigment pattern in response to social cues [5].

Early work to understand the genetic basis of variation in pigmentation focused on the analysis of mutant mice. Recently, fish have become an attractive model system due to their short generation times, large numbers of offspring, and the significant genetic resources available for some species. Zebrafish and medaka have been valuable models for identifying genes integral to pigment pattern formation via mutational analysis [6-9]. Additional work has connected these genes to variation in pigment phenotypes among species of cyprinid fishes [1]. Despite these advances, there is still much to be learned about the genetic basis of pigment patterning in natural populations.

Fishes display some of the most spectacular pigmentation observed in nature. Not only can a variety of colors be found, but also patterning including bars, stripes, spots, concentric rings, and blotches [10]. These pigment patterns are formed by a diversity of pigment cells derived from the neural crest [11]. While mammals and birds possess only the melanin- containing melanocytes, fish have been found to have three basic pigment cell types: black melanophores, containing melanin, yellow to orange xanthophores, containing carotenoid or pteridine derived pigments, and highly reflective iridophores, which contain guanine platelets [12]. Additional cell types have been identified in some fish, including blue cyanophores, red erythrophores, and white leucophores [12]. Positioning of these cells in relation to each other produces the varied colors and patterns seen in nature.

The cichlid fishes of East Africa provide an excellent example of fish pigment pattern diversity and evolution. Endemic radiations of cichlids have arisen in each of the three major Rift Valley lakes (Malawi, Victoria, and Tanganyika). Of the three radiations, Lake Malawi is of particular interest because it is thought that most of the 500+ species of cichlids in the lake have arisen over the last two million years [13]. Diversification of pigment patterns dominates the most recent stage in the radiation, which has been driven by sexual selection [14]. Despite the importance of pigmentation to cichlid speciation, surprisingly little has been done to identify the genes associated with the diverse color patterns in these fishes [15-17].

Because many of the species in Lake Malawi can be hybridized, it is possible to use a forward genetics approach to map genes underlying phenotypic diversity [18]. We previously analyzed an F_2_ hybrid cross that suggested only a small number of genes underlie pigmentation differences between two Lake Malawi African cichlids, *Metriaclima zebra* and *M*. *mbenjii*[19]. In the present study, we have identified several hundred single nucleotide polymorphisms (SNPs) by sequencing restriction site associated DNA markers (RADSeq). We genotyped these markers to construct a linkage map for the hybrid cross and identify quantitative trait loci (QTL) underlying the pigmentation traits. Finally, we used the marker sequences to anchor the *M*. *zebra* genome sequence assembly to the linkage map, in order to identify candidate genes within each QTL interval.

## Methods

### Phenotypes

**Figure 1:**
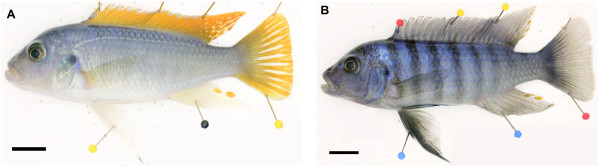
**F**_**0 **_**parents of the hybrid cross. A**) *M*. *mbenjii* male. Note the orange dorsal and caudal fins, plain blue body, and white pelvic fin. **B**) *M*. *zebra* male. Note the blue dorsal and caudal fins, barred body, and black pelvic fin.

An F_2_ hybrid cross was generated by crossing a single male *M*. *mbenjii* to a single female *M*. *zebra*. This resulted in a single F_1_ family that had a single male intercrossed to sibling females to produce the F_2_ offspring. While both male and female F_2_ offspring were produced, only sexually mature, dominant male F_2_ were analyzed. The two grandparent species differ in several aspects of male pigmentation. Male *M*. *mbenjii* have a light blue body with orange dorsal and caudal fins. Their pelvic fins are clear with an iridophore streak on the leading edge (Figure 1A). Male *M*. *zebra* have a light blue body with black bars and blue dorsal and caudal fins. Their pelvic fins have a melanophore streak on the leading edge. They also have dark cheeks and a black bar between the eyes (Figure 1B). While the xanthophores on the caudal fin of *M*. *mbenjii* are found in stellate form and overlap to form a uniform field, *M*. *zebra* often possess small punctate xanthophores on their caudal fins. The following quantitative phenotypes were collected from 160 F_2_ male fish: # of melanophores on scales from the dark body bars, # of melanophores on scales from the spaces between the bars, # of dorsal fin melanophores, # of caudal fin melanophores, # of pelvic fin melanophores, # of cheek melanophores, dorsal fin xanthophore area, caudal fin xanthophores area, pelvic fin xanthophores yellow score, gular xanthophores yellow score, and principal component one of a multivariate analysis of all ten traits. A detailed description of the measurement of these traits was published previously [19]. The reader is referred to Additional file 1 if they would like to access the phenotype data for all individuals used in the QTL study. All animal procedures were approved by the University of Maryland IACUC (Protocol no. R-10-73).

### Genotypes

SNPs were identified and genotyped via restriction site associated DNA sequencing (RAD-seq) [20]. Reduced representation DNA libraries were created using the protocol of Baird et. al 2011 [21]. Five libraries each containing multiplexed barcoded DNA for 32 individuals (160 F_2_ progeny total), were sequenced in separate lanes of an Illumina HiSeq 1000. The F_0_ grandparents were sequenced in an additional lane with 9 other individually barcoded samples. Reads were quality filtered by requiring Sanger quality scores of at least Q20 across 90% of the read. Reads were processed for individual barcodes and then assembled *de novo* into loci using the software pipeline Stacks v. 0.996 [22]. A minimum of 10 identical reads was required to create a “stack” in the parents. A minimum of 3 identical reads was required to create a stack in progeny individuals. One mismatch between loci was allowed when building the stacks “catalog”. Resulting loci that were differentially fixed between the grandparents, in Hardy-Weinberg equilibrium, and successfully genotyped in 100 or more F_2_ individuals were chosen for creating the linkage map.

Additional microsatellite markers for the putative sex locus and selected candidate genes were added by selecting previously described microsatellite primer sequences from GenBank or by designing new primers to microsatellites identified in the *M*. *zebra* genome browser (http://www.bouillabase.org). Information on these markers can be found in Additional file 2: Table S1.

### Linkage map construction and QTL scan

A linkage map was created using JOINMAP 3.0 [23]. The locus file consisted of genotypes at 798 SNPs and 37 microsatellites for 160 F_2_ male progeny derived from a single F_1_ family. The grouping module of JOINMAP assigned 834 of these markers to 22 linkage groups using a logarithm of odds (LOD) score of 5.0. We built a genetic map with the mapping module of JOINMAP, using the Kosambi mapping function, a recombination threshold of 0.450, and a jump threshold of 5.0. Linkage group numbers were assigned based on homology to the existing linkage groups of tilapia [24,25].

QTL were detected using R/qtl [26,27]. First, one thousand permutations were run using a Haley-Knott regression. The 5% significance level corresponded to an average LOD threshold of 4.1 for all phenotypes. We used a stepwise QTL detection algorithm that allowed for the detection of up to ten QTL for each phenotype, with the possibility of interactions. QTL intervals were further examined for significance by determining the Bayesian credible interval. Genes in these credible intervals were compiled using the gene annotations found on the *M*. *zebra* genome browser (http://www.bouillabase.org). Candidate genes were identified via literature searches using the gene names.

### Anchoring the *M*. *zebra* genome assembly

The locations of mapped markers on the *M*. *zebra* genome assembly version 0 were determined via BLAST. Assembly scaffolds were placed into anchored linkage groups if there were at least two markers from the same linkage group that blasted to that scaffold. The order and orientation of these scaffolds within each linkage group was then determined based on the BLAST location of markers relative to one another.

## Results and discussion

### RAD-tag sequencing

A total of 743,486,491 reads were produced from the 5 lanes of Illumina HiSeq for the 160 F_2_ progeny. 662,570,635 (89.1%) passed our Q20 filter. A total of 624,492,015 reads (94.3% of the filtered reads) were successfully processed for barcodes by Stacks. This corresponds to approximately 7.7 million reads per F_2_ progeny. After filtering and barcode processing, there were 7,535,127 reads for the F_0_ female and 18,850,602 reads for the F_0_ male used for the cross.

### Map construction and anchoring

We scored 834 genetic markers in 160 male F_2_ progeny from the *M*. *zebra* x *M*. *mbenjii* cross. The average coverage of each genotype SNP was 49.9x (range of 6.9x-201.7x) in the F_2_ progeny, 254.9x in the male F_0_, and 105x in the female F_0_. The average genotype completeness was 77% and the frequencies of each genotype class were 27.7% AA, 45.5% AB, and 26.9% BB (A designated for grandfather alleles and B for grandmother alleles). 60 individuals had between 0–100 genotypes missing, 42 individuals had 101–200 genotypes missing, 25 individuals had between 201–300 genotypes missing, 22 individuals had between 301–400 genotypes missing, 7 individuals had between 401–550 genotypes missing, and 4 individuals had greater than 500 genotypes missing. The average number of missing genotypes per individual was 182. High numbers of missing genotypes can be attributed to low coverage in some of the F_2_ individuals, with coverage ranging from 357,000 reads to 10,500,000 reads. We obtained a linkage map that contained 22 linkage groups and spanned over 1,933 cM. This agrees with previous work indicating that there are 22 chromosomes in cichlids [25]. Marker density was approximately one marker per 2.5 cM. Additional file 1 contains the information used to create the linkage map.

This linkage map was then used to order scaffolds of the *M*. *zebra* genome assembly. To be included in the anchored map, we required that scaffolds be anchored by at least two markers in the linkage map. We found 114 scaffolds (6.5 per linkage group) that met this criterion. The average size of these scaffolds was 3,918,467 bp for a total of 564,259,264 anchored bp. This represents 66.5% of the 848,776,495 bp *M*. *zebra* genome assembly. An additional 110 scaffolds had a single hit to a particular linkage group and were not included in the anchoring. If these single scaffolds were included in the anchoring, 92.3% of the assembly would become anchored. Additional file 3 provides the placement of scaffolds on the linkage groups.

### QTL scan and detection

**Figure 2:**
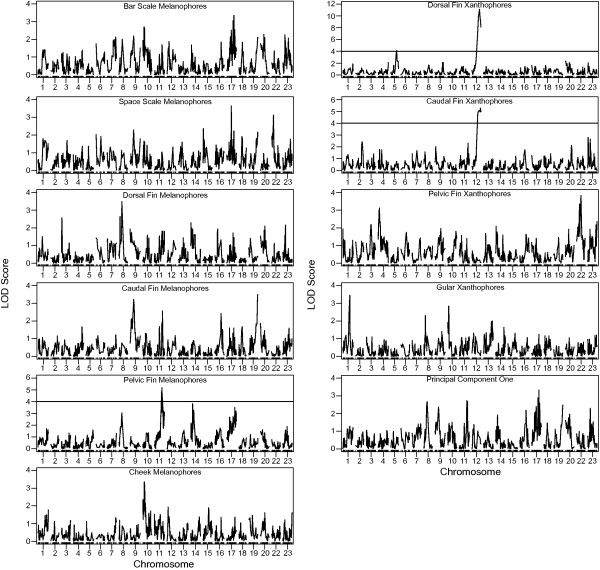
**Genome wide distribution of LOD scores for each phenotype examined.** Most traits fell below the 5% LOD significance threshold of 4.1, with the exception of pelvic fin melanophores, caudal fin xanthophores, and dorsal fin xanthophores.

A genome wide scan resulted in the identification of three QTL. Phenotypes with significant LOD scores included dorsal fin xanthophores, caudal fin xanthophores, and pelvic fin melanophores (Figure 2). The failure to detect QTL for the remaining pigmentation traits is most likely due to the small difference in the parental means for the other pigmentation traits. QTL were detected for the three traits for which parental means were 2.8 or greater standard deviations apart. The mapping population of 160 male F_2_ did not have enough power to detect QTL for traits for which the difference in parental means was smaller than 2.8 standard deviations.

### Dorsal fin xanthophores

**Figure 3:**
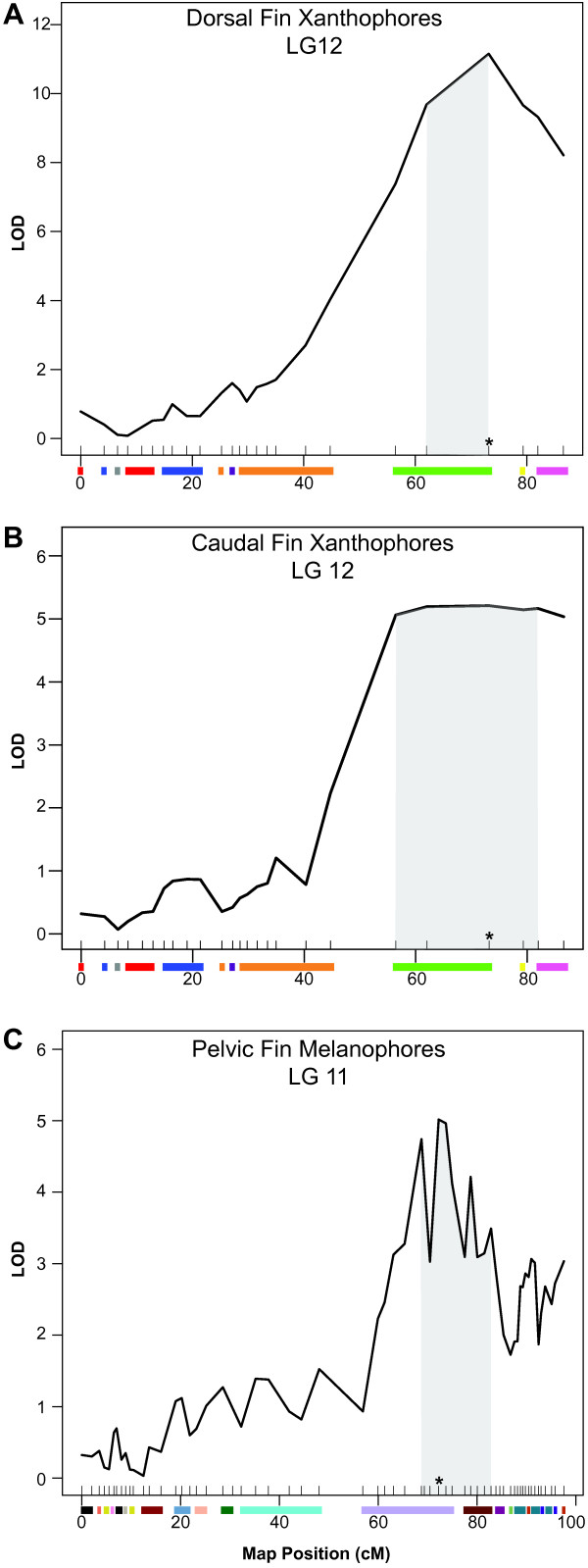
**QTL plots for each trait that exceed the significant LOD threshold.** Shaded area indicates the Bayesian credible interval. The colored bars on the x-axis represent different genomic scaffolds. **A**) Dorsal fin xanthophores on LG12. **B**) Caudal fin xanthophores on LG12. **C**) Pelvic fin melanophores on LG11.

**Figure 4:**
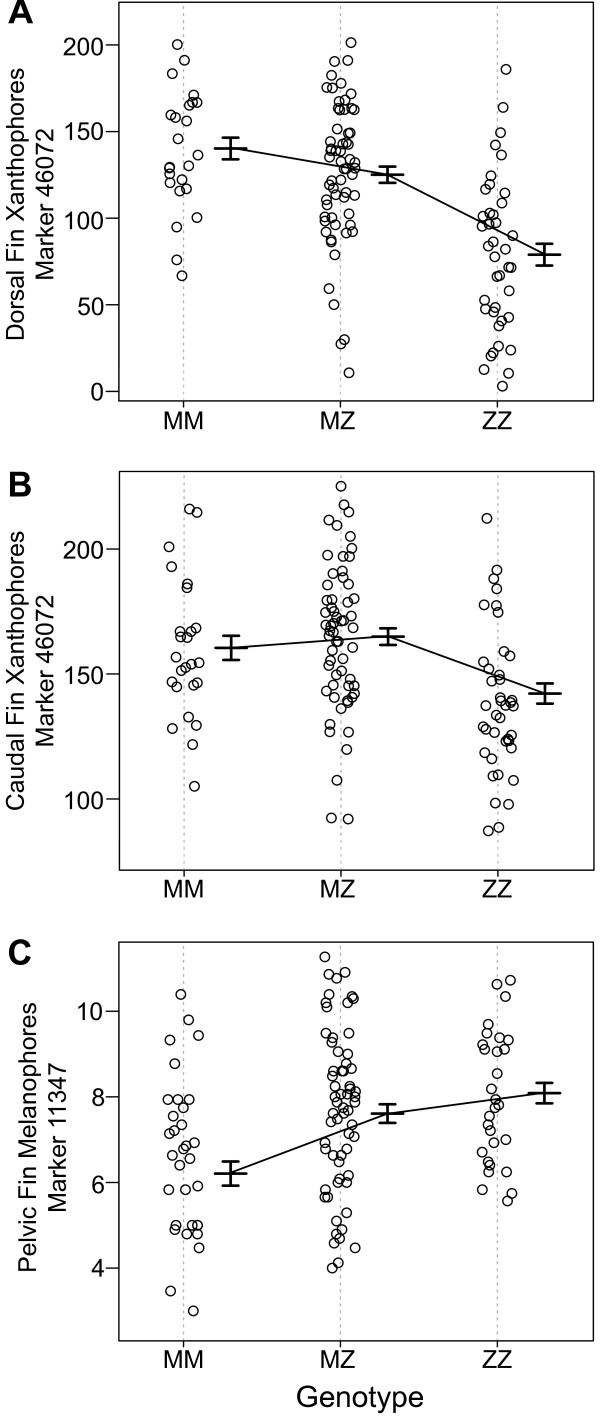
**Effect plots for each trait at the marker with the highest LOD score.** For each plot, “M” represents *M*. *mbenjii* alleles and “Z” represents *M*. *zebra* alleles. **A**) Dorsal fin xanthophores **B**) Caudal fin xanthophores **C**) Pelvic fin melanophores.

One QTL with a LOD score of 11.15 was detected for dorsal fin xanthophores on LG12 and explained 27.4% of the variance (Figure 3A). The Bayesian credible interval for this QTL spans from 62–73 cM along LG12. This matches the estimate from our previous work that a minimum of one QTL would be detected for this trait [19]. The effect plot for the marker with the highest LOD score shows incomplete dominance. Individuals with more orange on their dorsal fin possess at least one allele from the *M*. *mbenjii* grandfather (Figure 4A). The results of the effect plot are consistent with the inheritance of orange fins from the *M*. *mbenjii* grandfather [19].

Using the annotated genome assembly for *M*. *zebra*, we were able to identify candidate genes within the credible interval. Little work on the genetics of xanthophore development and carotenoid formation has been done in fishes. *Csf1r*, the most obvious candidate gene for xanthophores traits [8], is not present in the interval. Several other genes that might be involved in pigment cell development are present, including genes involved in vesicle formation, carotenoid synthesis, and cell aggregation (Additional file 4: Table S2). TRPM 6/1, a member of a gene family previously linked to pigment cell development in zebrafish, is located in the interval. However, this gene has only been demonstrated to be important in melanophore development. Fish possessing a mutation in this gene experience melanophore death, while the other pigment cell lineages (xanthophores and iridophores) appear to develop normally [28,29]. We do not consider TPRM6/1 a primary candidate gene for dorsal fin color since the difference between the grandparent fins appears to be a trade-off between the presence of xanthophores versus iridophores.

### Caudal fin xanthophores

One QTL with a LOD score of 5.21 was identified for caudal fin xanthophores on LG 12 in a region that overlaps with, but is broader than, the QTL region identified for dorsal fin xanthophores. The Bayesian credible interval spans from 56–81 cM along LG 12 (Figure 3B). Identification of a shared QTL is not surprising since our previous work indicated a strong correlation between these traits [19]. The QTL plot for caudal fin xanthophores is broader, possibly because this trait shows less variance in the caudal fin than it does in the dorsal fin. While the xanthophores in the caudal fin of *M*. *mbenjji* are found in stellate form and overlap to form a uniform field, *M*. *zebra* often possess small punctate xanthophores on their caudal fins.

Although the QTL region for dorsal fin and caudal fin xanthophores is shared, the percent variance explained for each trait is different, with 27.4% of the variance explained for the dorsal fin xanthophores, but only 14% of the variance explained for the caudal fin xanthophores. This difference could be due to the number of genes predicted to control each of these traits. While the Castle-Wright estimator predicted that one gene controls dorsal xanthophores, a minimum of three genes was predicted to control caudal fin xanthophores [19]. We were probably only able to detect one gene for caudal fin xanthophores due to the power limitations discussed previously.

Similar to the effect plot for dorsal xanthophores, individuals with more orange on their caudal fin possess at least one of the *M*. *mbenjii* grandfather’s alleles (Figure 4B). It should be noted that while the trait appears to show overdominance, the mean for the individuals homozygous for the *M*. *mbenjii* alleles and the mean for heterozygotes are not significantly different. Candidate genes considered for this region are the same as those considered for the dorsal fin xanthophores.

### Pelvic fin melanophores

One QTL with a LOD score of 5.01 was identified for pelvic fin melanophores, with a Bayesian credible interval spanning from 68–82 cM on LG 11 (Figure 3C). This QTL explains 13.4% of the variance for this trait. We previously estimated that this trait was controlled by a minimum of three genes. The relatively low power of our mapping population may explain why only one QTL was found for this trait. The jagged QTL curve for this trait may be due to a combination of high marker density and occasional missing genotypes.

Effect plots show that individuals with more melanophores on their pelvic fin are homozygous for the *M*. *zebra*’s grandmaternal alleles (Figure 4C). This is consistent with the fact that *M*. *zebra* males possess more melanophores on their pelvic fin compared to *M*. *mbenjii* males [19]. No obvious candidate genes were identified, however various genes in the interval play a role in human skin disease, interact with other genes involved in melanophore development, or are involved in vesicle formation, packaging, and trafficking (Additional file 4: Table S2).

## Conclusions

Our study confirms that RADSeq is an effective method for rapidly identifying SNPs and genotyping hybrid crosses. We were able to obtain a high density of markers throughout the genome, with one marker approximately every 2.5 cM. Using these makers, we were able to create a linkage map, and subsequently anchor 66.5% of the current *M*. zebra genome assembly. We were also able to identify QTL regions for three of the eleven pigmentation traits studied. One region on LG12 contained a shared QTL for dorsal and caudal fin xanthophores. A second region on LG11 contains a QTL for pelvic fin melanophores.

The number of QTL identified and the percent variance explained appears to be consistent with our previous work. For dorsal fin xanthophores, we predicted we would identify one gene controlling this trait. This trait was the one for which we identified the highest LOD score, had the highest percent variance explained, and had the narrowest QTL peak. The other pigment traits were predicted to be controlled by multiple genes. Not surprisingly, these traits had lower LOD scores, explained a smaller portion of the variance, and had broader QTL peaks. Despite our high marker density, the size of the mapping population limited our ability to narrow our QTL regions to less than 11 cM.

Finally, analysis of the predicted genes within our intervals showed several genes that could play a role in the development of pigmentation. None of them correspond to well-known zebrafish genes previously known to play a role in pigment cell development. Thus, these QTL represent an opportunity to learn something new about the genes underlying variation in pigmentation among fishes. We are particularly excited to have identified a major QTL contributing to xanthophore development, about which so little is known.

## Competing interests

The authors declare that they have no competing interests.

## Authors’ contributions

CTO helped to conceive of the study and participated in design, coordination, data collection, and analysis, and drafting of the manuscript. ACD helped to with data collection and analysis. MAC helped with data collection and analysis and drafting of the manuscript. TDK helped to conceive of the study and participated in design and coordination, and drafting of the manuscript. All authors read and approved the final manuscript.

## Supplementary Material

Additional file 1 **Markers_QTLAnalysis_Locations.xlsx QTL markers and their locations in the genome.** Columns B-L contain phenotype information for all investigated pigmentation traits for each F_2_ individual. Marker names are given in row 1, followed by the linkage group, position (centimorgans) on that linkage group, the scaffold and scaffold position (basepair) at which the marker is found is given beneath each marker name. The rows following provide the genotype for each F_2_ individual at that marker is also given. Click here for file

Additional file 2: Table S1 Non-RAD primer sequences. Click here for file

Additional file 3 **MZebra_anchoredmap.xlsx Anchored *M*. *zebra *map.** This file shows the linkage groups and the scaffolds contained within them. For each linkage group, markers that hit to the same scaffold are color-coded the same, with the exception of those with only one hit, which are color-coded light yellow. Linkage groups are separated by a black bar. Markers that were placed in the linkage group by JoinMap but excluded during the anchoring process are noted. Click here for file

Additional file 4: Table S2 Candidate genes for identified QTL regions. Click here for file
