# Supplementary material for: Mapping of pigmentation QTL on an anchored genome assembly of the cichlid fish, Metriaclima zebra
Source: BMC Genomics. 2013 Apr 27;14:287. doi: 10.1186/1471-2164-14-287 (PMC3691601; doi:10.1186/1471-2164-14-287)
Supplement: Additional file 2: Table S1 — Non-RAD primer sequences. [file 1471-2164-14-287-S2.docx]

| **Table S1.** Non-RAD primer sequences | | | |
| --- | --- | --- | --- |
| **Primer Name** | **Forward Primer Sequence** | **Reverse Primer Sequence** | **Genomic Location-Scaffold:Base** |
| Spindly | CAGGTGGTTGGTTTTCTTGG | TCTCTCAGACGCTCTGCATC | 34:228462 |
| CSF1R | TGAACAGGTTGCTTACGCTCT | TGCTGTCTCTTGGTGGTGTT | 198:829661 |
| MZ28416 | CCATCAGCCAATCAGCTACA | GGAAGCCCCAGTGTCAGATA | 58:1973203 |
| usat14168 | TGCTCAACACAGCTAAGCAAA | AGAGGGATTAAGGCGGATGT | 18:1030085 |
| usat9538 | CACAAGGTCCAATTCCATCC | TTTTGCTTCCACCACGATAA | 10:1042581 |
| MZ10190 | CTGTCTTCGCGTGGTGACTA | CGGTGTAATAAACACCCACCA | 10:9603154 |
| CADH | GGACGGTTCTTTCTTGGTCA | ACTTCCTGCCAGTGTTGGAC | 273:205421 |
| usat49371 | GGGATTAACGCTGCCTCATA | AGCCTCATTCGCAGAGTCAT | 202:914425 |
| MITF | TGCCGACTCTAAGAGCACCT | GGGCAAGCCATTGTTCAGTA | 130:258851 |
| MZ29472 | GCCCATTGATGTGGCTTAAA | GCTGCCTTTCCAGCTATTCA | 63:2335426 |
| MZ1012 | GAGCACATTGACTGGTGTGC | ATTCTGTAGGCCCATTGCAG | 0:13037865 |
| MZ371 | AAGGTAATTTGCAGGGAGCA | CCAGGTTGATGGATGGAAGT | 0:5124400 |
| Labeo2016 | CCCAAATTGTTCATCACCAT | CCCTGATCTTTGTCAAACCAG | 21:2801036 |
| MZ33953 | CTATTGAGCCCCACATTGCT | TGTGCGTGGAGCTTTGTTAG | 84:1877802 |
| MZ31651 | TTAACAGTGTAAGCGCAAACG | AAACTCCACCGCTGTCAAAC | 73:2547017 |
| Somato17 | AGAGCCATGTGCAATCAACA | TGACTTGAAAGATGGGCTGA | 8:9213226 |
| Somato20 | GGTCTGGTGGCCAGGTAGTA | CCTCCTGCTCTGTTCCTCAG | 8:9261286 |
| MZ27256 | ATACATGCAGCGAGCACAAA | TGGGCTGGTATGAAACACAA | 54:1524636 |
| MZ30207 | TTTTGGTGTCTGTGCTTTGG | TAATTCCTCGCCCAATGTTC | 67:547222 |
| MZ52420 | CCCACAGTTCTGGACAACCT | GAGCTGGCCAACTACTACGC | 272:154037 |
| MZ37607 | GCACACGTCCTCTCTTCCTC | AAGAGGAAAGCACTGGGAGA | 103:1963623 |
| MZ13482 | TGTCACACTGAGAGAAGGAAGAGA | TAAGAACGGTGCCTGTGTTG | 16:5485770 |
| MZ13525 | TCCGTCCCTCTCTGTCTACC | GACTGAAAGCAGGAGGCTGT | 16:6011743 |
| MAP | AACCAATCAATGAGCTGGAA | GTGGAACTCACTGGCACACA | 49:532113 |
| SL9A7 | TCAAGAGTCCAAGTGACACCA | AGCCTCAGCAGAAGAACCAG | 49:462833 |
| Syntaxin | AGCAGTGTGAGGACGGAGTT | TTCGTTAGTCAACGTGTGTGTG | 49:732084 |
| Bloc1S2 | TCCACTGATGAGGTGAGGTT | ACAATGGCCAAAGGACAAAA | 49:2903493 |
