# Supplementary material for: Mapping of pigmentation QTL on an anchored genome assembly of the cichlid fish, Metriaclima zebra
Source: BMC Genomics. 2013 Apr 27;14:287. doi: 10.1186/1471-2164-14-287 (PMC3691601; doi:10.1186/1471-2164-14-287)
Supplement: Additional file 4: Table S2 — Candidate genes for identified QTL regions. [file 1471-2164-14-287-S4.docx]

| **Table S2.** Candidate genes for identified QTL regions | | | |
| --- | --- | --- | --- |
| **QTL Region** | **Gene** | **Function** | **Reference** |
| Dorsal and Caudal Xanthophores | GAPDV1-GTPase-activating protein and VPS9 domain-containing protein 1 | Interacts with RAB5 as a GEF and coordinates vesicle uncoating. | Stenmark H: **Rab GTPases as coordinators of vesicle traffic**. Nat *Rev Mol Cell Bio* 2009, **10**:513-525. |
| Dorsal and Caudal Xanthophores | STAR-Steroidogenic acute regulatory protein | Thought to be important in carotenoid binding and deposition. | Walsh N, Dale J, McGraw KJ, Pointer MA, Mundy, NI: **Candidate genes for carotenoid coloration in vertebrates and their expression profiles in the carotenoid-containing plumage and bill of a wild bird.** *Proc R Soc B* 2012, **279**: 58-66. |
| Dorsal and Caudal Xanthophores | TRPM6/1-Transient receptor potential cation channel subfamily M member 6/1 | Member of a gene family that has been previously linked to melanophore mutations in zebrafish. | Iuga AO, Lerner EA: **TRP-ing up melanophores: TRPM7, melanin synthesis, and pigment cell survival**. *J Invest Dermatol* 2007, **127**: 1855-1856. |
| Dorsal and Caudal Xanthophores | VP33A-Vacuolar protein sorting-associated protein 33A | Thought to be involved in melanosome biogenesis; causes pigment dilution mutant in mice | Suzuki T, Oiso N, Gautam R, Novak EK, Panthier JJ, Suprabha PG, Vida T, Swank RT, Spritz RA: **The mouse organellar biogenesis mutant buff results from a mutation in *Vps33a*, a homologue of yeast *vps33* and *Drosophilia* carnation.** *PNAS* 2003, **100**: 1146-1150. |
| Dorsal and Caudal Xanthophores | RP3A-Rabphilin-3A | Effector of Rab27. Rab27a mutations in humans and mice results in melanosome transport defects. | Chavas LMG, Ihara K, Kawasaki M, Torii S, Uejima T, Kato R, Izumi T, Wakatsuki S: **Elucidation of Rab27 recruitment by its effectors: structure of Rab27a bound to Exophlin4/Slp2-a.** *Structure* 2008, **16**: 1468-1477. |
| Dorsal and Caudal Xanthophores | ADRB2-Beta-2-adernergic receptor | Causes pigment dispersion of melanophores. | Morishita F, Katayama H, Yamada K: **Subtypes of beta adrenergic receptors mediating pigment dispersion in chromatophores of the medaka, *Oryzias latipes***. *Comp Biochem Physiol* 1985, **81C:** 279-285. |
| Dorsal and Caudal Xanthophores | AAK1-AP2-associated protein kinase 1 | Enchances stabilization of AP2 with plasma membrane and its derived vesicles. | Stenmark H: **Rab GTPases as coordinators of vesicle traffic**. *Nat* *Rev Mol Cell Bio* 2009, **10**:513-525. |
| Dorsal and Caudal Xanthophores | BCDO2-Beta-carotene 9’;10’-oxygenase | Cleaves colorful carotenoids to colorless apocarotenoids; reduction of expression causes yellow skin in chickens | Eriksson J, Larson G, Gunnarsson U, Bed’hom B, Tixier-Boichard M, Stromstedt L, Wright D, Jungerius A, Vereijken A, Randi E, Jensen P, Andersson L: **Identification of the *Yellow Skin* gene reveals a hybrid origin of the domestic chicken**. *PLoS Genet* 2008, **4**: e1000010. |
| Dorsal and Caudal Xanthophores | ARRD3-Arrestin domain-containing protein 3 | Can regulate β2-adernergic receptors, which in turn can cause pigment dispersion | Nabhan JF, Pan H, Lu Q: **Arrestin domain-containing protein 3 recruits the NEDD4 E3 ligase to mediate ubiquitination of the β2-adernergic receptor.** *EMBO reports* 2010, **11:** 605-611  Morishita F, Katayama H, Yamada K: **Subtypes of beta adrenergic receptors mediating pigment dispersion in chromatophores of the medaka, *Oryzias latipes***. *Comp Biochem Phys C* 1985, **81**: 279-285. |
| Dorsal and Caudal Xanthophores | IQGA1-RAS GTPase-activating like protein IQGAP1 | Expressed by human keratinocytes | Presslauer S, Hinterhuber G, Cauza K, Horvat R, Rappersberger K, Wolff K, Foedinger D: **RasGAP-like Protein IQGAP1 is expressed by human keratinocytes and recognized by autoantibodies in association with bullous skin disease.** *J Invest Dermatol* 2003, **120**: 365-371. |
| Pelvic Melanophores | POU domain-Class3; transcription factor 1 | Also known as BRN2. Thought to be involved in melanocyte differentiation and growth. | Cook AL, Boyle GM, Leonard JH, Parsons PG, Sturm RA: **BRN2 in melanocytic cell development, differentiation, and transformation.** In *From Melanocytes to Melanoma: the Progression to Malignancy.* Edited by Hearing VJ and Leon SPL. New Jersey: Humana Press; 2006: 149-167. |
| Pelvic Melanophores | CXB5-Gap junction-beta 5 protein | Also known as Connexin 31.1. Shown to be expressed in human skin. | Aasen T, Kelsell DP: **Connexins in skin biology.** In *Connexins*. Edited by Harris AL and Locke D. New Jersey: Human Press; 2009: 307-321.  Scemes E, Spray DC, Meda P: **Connexins, pannexins, innexins: novel roles of “hemi-channels”.** *Pflugers Arch* 2009, **457**: 1207-1226. |
| Pelvic Melanophores | MEOX2-Homeobox protein MOX-2 | Blocks Pax3-DNA interactions; Pax3 involved in melanogenesis | Kubid JD, Young KP, Plummer RS, Ludvik AE, Lang D: **Pigmentation PAX-ways: the role of Pax3 in melanogenesis, melanocyte stem cell maintenance, and disease.** *Pigment Cell Melanoma Res* 2008, **21**: 627-645. |
| Pelvic Melanophores | DGKB-Diacylglycerol kinase beta | Can regulate levels of DAG; increased DAG increased melanin levels in cultured human melanocytes | Martelli AM, Bortul R, Tabellini G, Bareggi R, Manzoli L, Narducci, P, Cocco L: **Diacylglycerol kinases in nuclear lipid-dependent signal transduction pathways.** *Cell Mol Life Sci* 2002, **59**: 1129-137.  Park HY, Lee J, Gonzalez S, Middlekamp-Hup MA, Kapasi S, Peterson S, Gilchrest BA: **Topical application of a Protein Kinase C inhibitor reduces skin and hair pigmentation.** *J Invest Dermatol* 2004, **122:** 159-166. |
| Pelvic Melanophores | ARF3-ADP-ribosylation factor 3 | Could possibly be involved in vesicle formation pathway. | Boman AL, Zhang C, Zhu X, Kahn, RA: **A family of ADP-ribosylation factor effectors that can alter membrane transport through the *trans*-Golgi**. *Mol Biol Cell* 2000, **11**: 1241-1255. |
